# Targeting Cancer Mitochondria by Inducing an Abnormal Mitochondrial Unfolded Protein Response Leads to Tumor Suppression

**DOI:** 10.7150/ijms.95624

**Published:** 2024-05-05

**Authors:** Baoxiao Wang, Wenjun Chen, Qiqi Huang, Ye Chen, Yajing Wang

**Affiliations:** 1 Department of Otolaryngology, Head and Neck Surgery, Sun Yat-sen Memorial Hospital, Sun Yat-sen University, Guangzhou, China.; 2 School of Medicine, Southern University of Science and Technology, Shenzhen, Guangdong, China.

**Keywords:** nasopharyngeal carcinoma, mitochondrial unfolded protein response, mitochondria, cisplatin, oxidative stress

## Abstract

The mitochondrial unfolded protein response (UPRmt) is a pivotal cellular mechanism that ensures mitochondrial homeostasis and cellular survival under stress conditions. This study investigates the role of UPRmt in modulating the response of nasopharyngeal carcinoma cells to cisplatin-induced stress. We report that the inhibition of UPRmt via AEB5F exacerbates cisplatin cytotoxicity, as evidenced by increased lactate dehydrogenase (LDH) release and apoptosis, characterized by a surge in TUNEL-positive cells. Conversely, the activation of UPRmt with oligomycin attenuates these effects, preserving cell viability and reducing apoptotic markers. Immunofluorescence assays reveal that UPRmt activation maintains mitochondrial membrane potential and ATP production in the presence of cisplatin, countering the rise in reactive oxygen species (ROS) and inhibiting caspase-9 activation. These findings suggest that UPRmt serves as a cytoprotective mechanism in cancer cells, mitigating cisplatin-induced mitochondrial dysfunction and apoptosis. The data underscore the therapeutic potential of modulating UPRmt to improve the efficacy and reduce the side effects of cisplatin chemotherapy. This study provides a foundation for future research on the exploitation of UPRmt in cancer treatment, with the aim of enhancing patient outcomes by leveraging the cellular stress response pathways.

## Introduction

Nasopharyngeal carcinoma (NPC) emerges as a significant malignancy, distinguished by its unique epidemiological distribution, predominantly in regions such as Southeast Asia, North Africa, and Southern China 1, 2. Intrinsically linked to Epstein-Barr virus (EBV) infection, NPC's etiology is deeply intertwined with this viral association 3, 4. From a therapeutic perspective, the management of NPC necessitates a multi-faceted approach, integrating radiotherapy, chemotherapy, and occasionally, surgical intervention. The NPC's distinctive anatomical positioning within the nasopharynx presents a unique set of challenges for treatment delivery, requiring the implementation of specialized modalities like intensity-modulated radiotherapy to optimize treatment outcomes while minimizing collateral damage 5-7.

In the contemporary landscape of NPC research, the emphasis has shifted towards precision medicine, seeking to exploit targeted therapies and immunotherapies to enhance treatment efficacy and patient outcomes. Concurrently, a growing body of research is focusing on understanding the intricacies of the tumor microenvironment, immune evasion strategies, and molecular profiling, fostering the development of personalized treatment regimens for NPC patients 8, 9. NPC manifests itself as a complex interplay of genetic, environmental, and viral factors, with EBV infection playing a central role, specifically through the expression of viral proteins that disrupt standard cellular processes and immune surveillance 10. Genetic susceptibility is also a critical component, with variations in genes associated with immune regulation, cell cycle control, and DNA repair influencing an individual's susceptibility to NPC 11. Environmental triggers, such as exposure to certain carcinogens like nitrosamines from preserved foods or tobacco smoke, further exacerbate the disease onset. NPC's pathogenesis, with its intricate crosstalk between viral, genetic, and environmental contributors, underscores the complexity of this malignancy 7, 12. A thorough understanding of these mechanisms at the molecular level is imperative to devise targeted therapies and precision medicine strategies effectively to combat nasopharyngeal carcinoma.

Mitochondria stand as integral arbiters in the proliferation, invasion, and metastasis of nasopharyngeal carcinoma (NPC) 13, 14. These organelles, renowned for their role in energy production via oxidative phosphorylation, also serve as pivotal regulators of cell survival, apoptosis, and signaling pathways instrumental in tumor progression 15. In the NPC landscape, mitochondria with compromised functionality foster tumor growth by manipulating metabolic pathways, accommodating heightened energy demands, and facilitating resistance to cell death 16, 17. Additionally, mitochondrial dynamics, encompassing fusion and fission processes, influence cell migration and invasion by modulating cytoskeletal dynamics and cellular motility 18-20. The interplay between mitochondria and other cellular compartments, such as the endoplasmic reticulum and nucleus, bolsters the invasive and metastatic potential of NPC cells. Disruptions in mitochondrial functions can amplify NPC tumor aggressiveness, accelerating their dissemination to distal sites 21-23. Deciphering the intricate dynamics between mitochondrial activity, metabolism, and signaling pathways is vital to unveiling novel therapeutic targets and devising strategies to thwart tumor growth, invasion, and metastasis in NPC patients.

The mitochondrial unfolded protein response (UPRmt) is a conserved stress response pathway, safeguarding mitochondrial proteostasis by orchestrating the expression of chaperones, proteases, and other factors implicated in protein folding and quality control within the mitochondria 24, 25. Upon exposure to a spectrum of stressors, such as oxidative damage, protein misfolding, or metabolic shifts, UPRmt is activated to reinstate mitochondrial homeostasis and foster cell survival 26, 27. Dysregulation of UPRmt has been implicated across a range of human diseases, including neurodegenerative disorders, metabolic disorders, and cancer, underscoring its critical role in maintaining mitochondrial functionality and cellular health 28-30. Understanding the mechanisms underpinning UPRmt activation and its subsequent impact on cellular physiology holds significant therapeutic implications, paving the way for the development of novel therapeutic strategies targeting mitochondrial proteostasis in disease contexts. Our study seeks to explore the influence of the mitochondrial unfolded protein response in the context of NPC growth, proliferation, and apoptosis.

## Methods

### Cell lines

The SUNE-1 cell line, procured from ATCC (Manassas, VA, USA), were propagated under controlled conditions. The medium of choice was DMEM, fortified with 10% fetal bovine serum, and the cells were sustained in an incubator set at 37°C with a 5% CO2 atmosphere. Oxidative stress was induced in the SUNE-1 cells through the introduction of hydrogen peroxide at a concentration of 0.3mM into the medium, with a duration of exposure of 6 hours. Concurrently, the SUNE-1 cells were also subjected to cisplatin (5mM) incubation. To modulate the mitochondrial unfolded protein response within the SUNE-1 cells, they were administered with either oligomycin (Catalog No. 579-13-5, MedChemExpress; 0.5mM) to stimulate the response, or AEBSF (Catalog No. S7378, Selleckchem, 2 mM) to suppress it.

### ELISA

Utilizing ELISA kits procured from R&D Systems, we quantified LDH concentrations in the medium and assessed the enzymatic activities of caspase-9 and caspase-3. Moreover, intracellular ATP production was ascertained with the same kits. All experimental protocols were meticulously executed in alignment with the manufacturer's guidelines.

### Immunofluorescence analysis

Cells underwent fixation using 4% paraformaldehyde, followed by permeabilization on ice utilizing a 0.5% Triton X-100 solution for a duration of 10 minutes. A subsequent blocking step was carried out for 60 minutes in 5% bovine serum albumin. Thereafter, cells were exposed to the primary antibody, in a solution constituted by 1% BSA-PBS and 0.1% Triton X-100. This incubation took place overnight at 4°C, in a light-protected environment, devoid of agitation. Subsequent to washing, cells were subjected to incubation with a secondary antibody for a period ranging from 1 to 2 hours at room temperature, along with Hoechst (1:1000) for a quarter of an hour.

### Real-time quantitative PCR

The procedure for RNA extraction from the cellular samples was carried out employing the Total RNA Kit II. The process of reverse transcription was initiated utilizing the PrimeScript RT Master Mix. The subsequent qPCR evaluation was facilitated by TB Green Premix Ex Taq II, and the reactions were performed on an Applied Biosystems' QuantStudio 7 Flex Real-Time PCR System (US, California based). The CT values garnered were set to a standard relative to βactin, and the differential expression levels were computed via the 2^-ΔΔCT^ methodology.

### TUNEL (terminal deoxynucleotidyl transferase dUTP nick end labeling) staining and efferocytosis analysis

In the context of scrutinizing apoptotic cells within atherosclerotic lesions, the In Situ Cell Death Detection Kit was employed in adherence to the guidelines provided by the manufacturer. The comprehensive count of apoptotic cells was then ascertained by quantifying the cells labeled with TUNEL (Terminal deoxynucleotidyl transferase dUTP nick end labeling). This procedure, in conjunction with efferocytosis analysis, provided a holistic view of the cellular landscape within atherosclerotic lesions.

### Western blotting assay

In the process of Western blotting analysis, cells were subjected to protein extraction and subsequent homogenization in a frigid lysis buffer. Following this, the homogenate was processed through centrifugation at 12,000 gravitational units for a quarter of an hour at a temperature of 4 °C, resulting in a supernatant that was duly collected. The protein content was then quantified spectrophotometrically utilizing a BCA kit (Beyotime, Cat#P0009, Shanghai, China). Subsequently, lysates underwent separation through 10% SDS-PAGE and were transferred onto polyvinylidene fluoride membranes. To block non-specific binding sites, a 5% BSA solution was employed for a duration of two hours at ambient temperature. The membranes were then subjected to incubation with primary antibodies in a 4 °C environment overnight. Upon completion of this step, the membranes were cleansed with Tris-buffered saline complemented with Tween 20, followed by a two-hour incubation period at room temperature with peroxidase-conjugated secondary antibodies (ABclonal, Cat#AS014). Visualization and quantification of the Western blot bands were accomplished using the ImageJ software (National Institutes of Health), with band intensity measurements being normalized to the internal control, as per previously established methods. This entire experimental procedure was executed independently on at least three separate occasions.

### Mitochondrial membrane potential detection and CCK-8 assay

The exploration of mitochondrial membrane potential, a critical indicator of cellular vitality, is achieved through a method that capitalizes on the properties of a specific fluorescent dye, JC-1. Intracellular JC-1 accumulation occurs in direct response to mitochondrial membrane potential, paving the way for subsequent analysis. Post-incubation, cells undergo a thorough rinse, followed by assessment via fluorescence microscopy. A decline in dye retention, indicative of mitochondrial depolarization, manifests as a color transition from red to green in the fluorescence spectrum.

In parallel, the CCK-8 assay serves as a potent tool in the evaluation of cell viability and proliferation. The procedure commences with the introduction of the CCK-8 solution to cell cultures, succeeded by a designated incubation duration. Cellular dehydrogenases trigger the reduction of the CCK-8 solution, culminating in the formation of a water-soluble formazan dye. The quantity of this dye is directly reflective of the living cell count. The final stage of the assay involves a spectrophotometric measurement of the formazan dye's absorbance at a specified wavelength. An increase in absorbance corresponds to a greater number of viable and actively proliferating cells.

### Mitochondrial ROS determination

The generation of reactive oxygen species (ROS) within cells was quantified utilizing the MitoSOX Red Mitochondrial Superoxide Indicator, supplied by Yeasen Biotechnology, Shanghai, China (Product Code: 40778ES50), as per the guidelines provided by the manufacturer. In brief, the mitochondria were subjected to MitoSOX staining (5 µM concentration) for a duration of 30 minutes within a humidified incubator maintained at 37°C and an atmosphere comprising 5% CO_2_. Post staining, the cells were washed and fixed, succeeded by nuclear staining using DAPI, executed as per the manufacturer's protocol. Subsequently, the DAPI-counterstained slides were examined under a fluorescence microscope. The quantification of the fluorescence was carried out using ImageJ software, a tool developed by the National Institutes of Health.

### Statistical analysis

Data representation follows the format of mean ± SEM. The chosen tools for statistical computations were GraphPad Prism (version 9) and SPSS software (version 22). The Shapiro-Wilk test was the default test for normality evaluation unless stated otherwise. In scenarios involving multiple groups, parametric data was processed using one-way ANOVA with the Bonferroni post hoc test. For non-parametric data, the Kruskal-Wallis test paired with Dunn's post hoc analysis was implemented. For comparisons between two conditions across groups, two-way ANOVA with Bonferroni post hoc correction was employed. Each experiment's specific statistical methodology is elaborated in the associated figure legends. The threshold for statistical significance was determined at P < 0.05.

## Results

### The suppression of mitochondrial Unfolded Protein Response (UPRmt) impairs cell viability and augments cancer apoptosis

**Figure 1:**
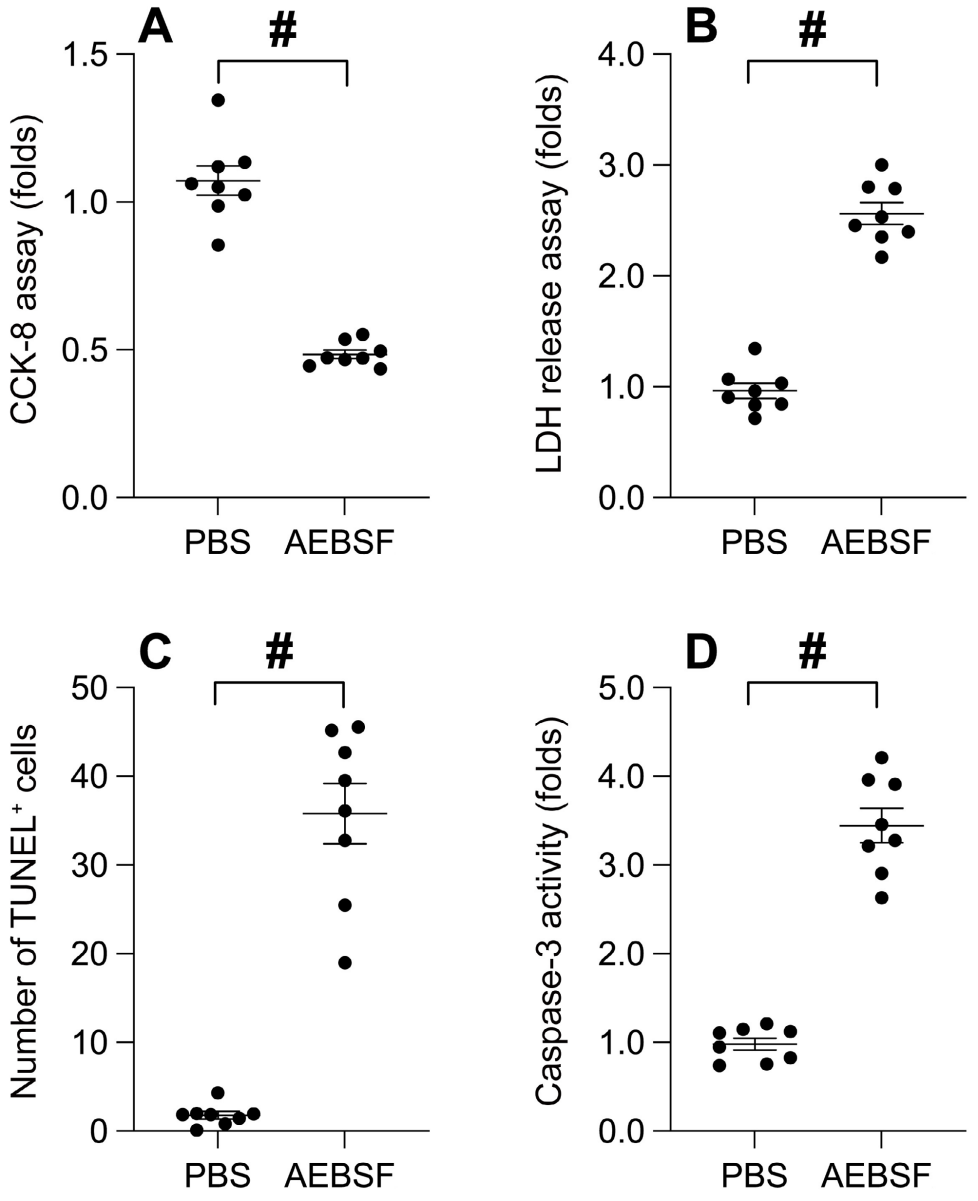
** The Suppression of Mitochondrial Unfolded Protein Response (UPRmt) Impairs Cell Viability and Augments Cancer Apoptosis. A.** SUNE-1 cells were treated with AEBSF to inhibit the activity of mitochondrial unfolded protein response (UPRmt). Then, cell viability was determined by CCK-8 assay. **B.** LDH release assay was measured via an ELISA kit. **C.** The number of TUNEL positive cells was evaluated by immunofluorescence. **D.** ELISA kit was used to determine the activity of caspase-3. #p<0.05.

To decipher the role of UPRmt in the survival of nasopharyngeal carcinoma, we employed an UPRmt inhibitor to treat SUNE-1 cells, a nasopharyngeal carcinoma cell line. We then evaluated cell viability using the CCK-8 assay. As depicted in Figure 1A, the application of AEBSF conspicuously curtailed cell viability compared to the control group. Concurrently, we noted an upsurge in LDH release into the medium in the aftermath of AEBSF treatment (Figure 1B). Additionally, TUNEL staining unveiled a marked increase in the count of apoptotic SUNE-1 cells post-AEBSF treatment compared to the PBS group (Figure 1C). Utilizing an ELISA kit, we observed a swift surge in caspase-3 activity following AEBSF exposure (Figure 1D). Collectively, these findings affirm that UPRmt inhibition undermines cell viability and accelerates cancer apoptosis.

### UPRmt inhibition precipitates mitochondrial dysfunction

**Figure 2:**
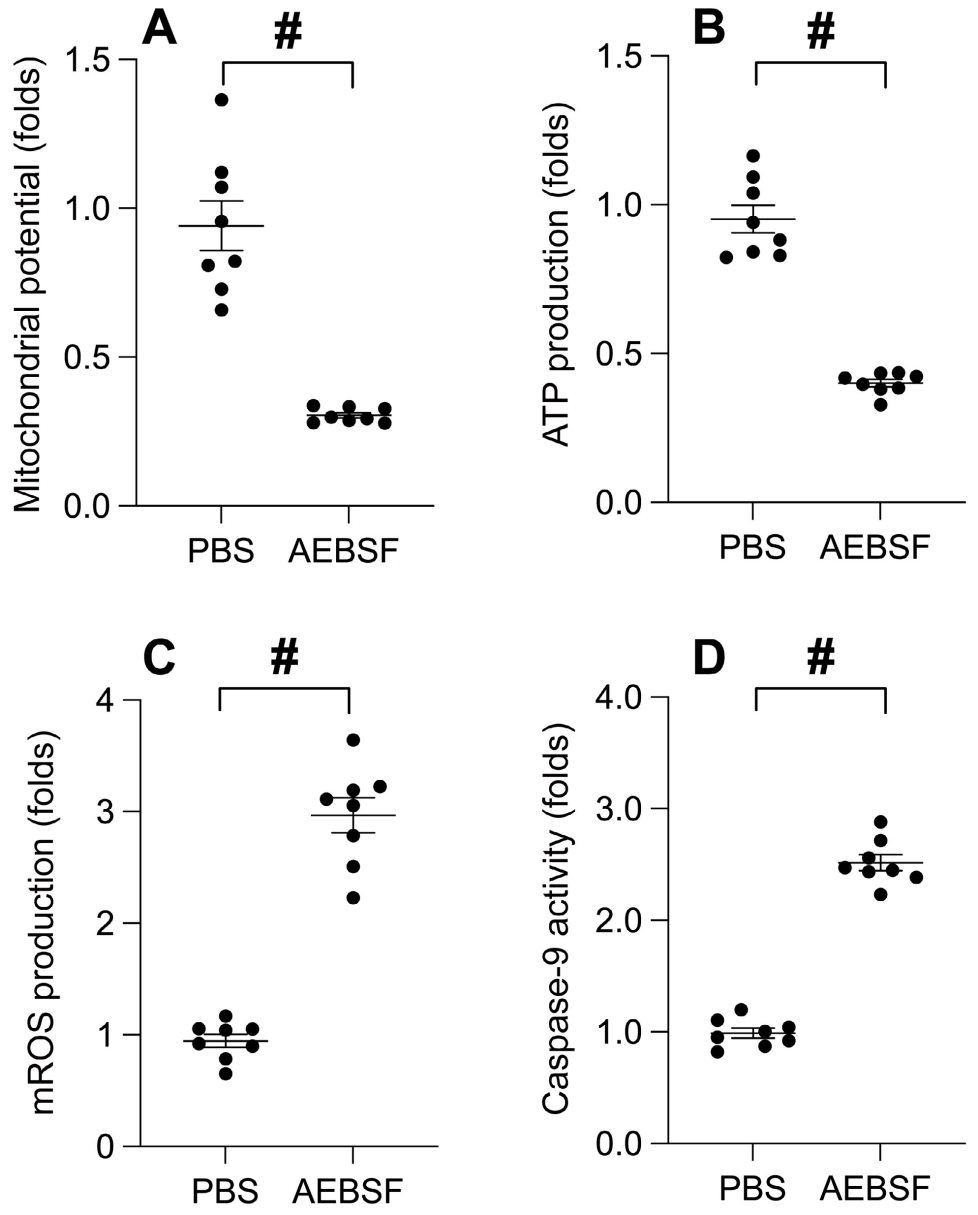
** UPRmt Inhibition Precipitates Mitochondrial Dysfunction. A.** SUNE-1 cells were treated with AEBSF to inhibit the activity of mitochondrial unfolded protein response (UPRmt). Then, mitochondrial potential was determined by immunofluorescence. **B.** ATP production was measured through ELISA. **C.** Mitochondrial ROS were measured by immunofluorescence. **D.** ELISA kit was used to determine the activity of caspase-9. #p<0.05.

To probe the interplay between UPRmt and mitochondrial functionality, we employed an immunofluorescence assay to gauge the mitochondrial membrane potential. Upon AEBSF exposure, we observed a substantial decline in the mitochondrial potential (Figure 2A). Simultaneously, the ATP concentration in SUNE-1 cells exhibited a pronounced reduction post-AEBSF treatment (Figure 2B). In stark contrast, the generation of mitochondrial reactive oxygen species (ROS) experienced a significant surge in the presence of AEBSF (Figure 2C). Moreover, the activation of Caspase-9, a recognized marker of mitochondrial apoptosis, was pronounced post-AEBSF treatment compared to the PBS group (Figure 2D). These findings unequivocally substantiate a link between UPRmt inhibition and the onset of mitochondrial dysfunction.

### UPRmt activation attenuates oxidative stress-induced cancer apoptosis

**Figure 3:**
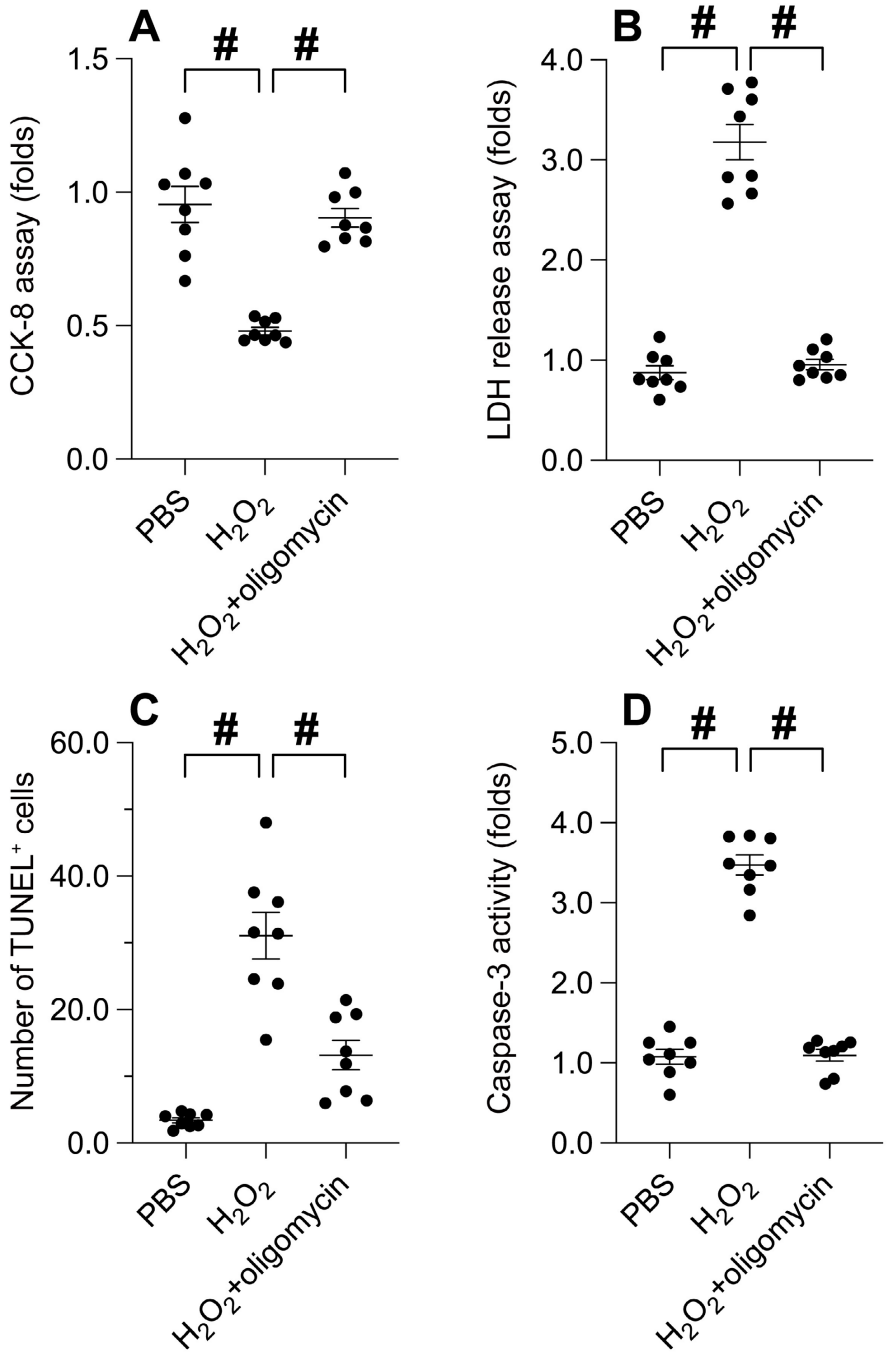
** UPRmt Activation Attenuates Oxidative Stress-Induced Cancer Apoptosis. A.** SUNE-1 cells were treated with hydrogen peroxide (0.3mM) to induce oxidative stress. Then, cell viability was determined by CCK-8 assay. **B.** LDH release assay was measured via an ELISA kit. **C.** The number of TUNEL positive cells was evaluated by immunofluorescence. **D.** ELISA kit was used to determine the activity of caspase-3. #p<0.05.

Our previous findings illuminate the cardinal role of UPRmt in preserving cancer cell viability and ensuring mitochondrial integrity. Consequently, we endeavored to explore whether UPRmt activation serves as a protective mechanism for cancer cells in adverse environments. SUNE-1 cells were subjected to hydrogen peroxide at a concentration of 0.3 mM for a duration of 6 hours. Subsequently, cell viability and apoptosis markers were re-assessed. As illustrated in Figure 3A-B, hydrogen peroxide treatment instigated a significant decline in cell viability and an upswing in LDH release compared to the PBS group. Intriguingly, these effects were notably mitigated by oligomycin, an UPRmt agonist. Furthermore, TUNEL staining revealed an increase in the count of TUNEL-positive cells in response to hydrogen peroxide, an effect counterbalanced by oligomycin treatment (Figure 3C). The activation of caspase-3 was evident in cells exposed to hydrogen peroxide; however, this effect was abrogated in SUNE-1 cells treated with oligomycin (Figure 3D). Collectively, these findings suggest that UPR^mt activation buffers cancer cell viability and obstructs apoptosis in the face of oxidative stress.

### UPRmt activation shields mitochondria from oxidative stress-induced damage

**Figure 4:**
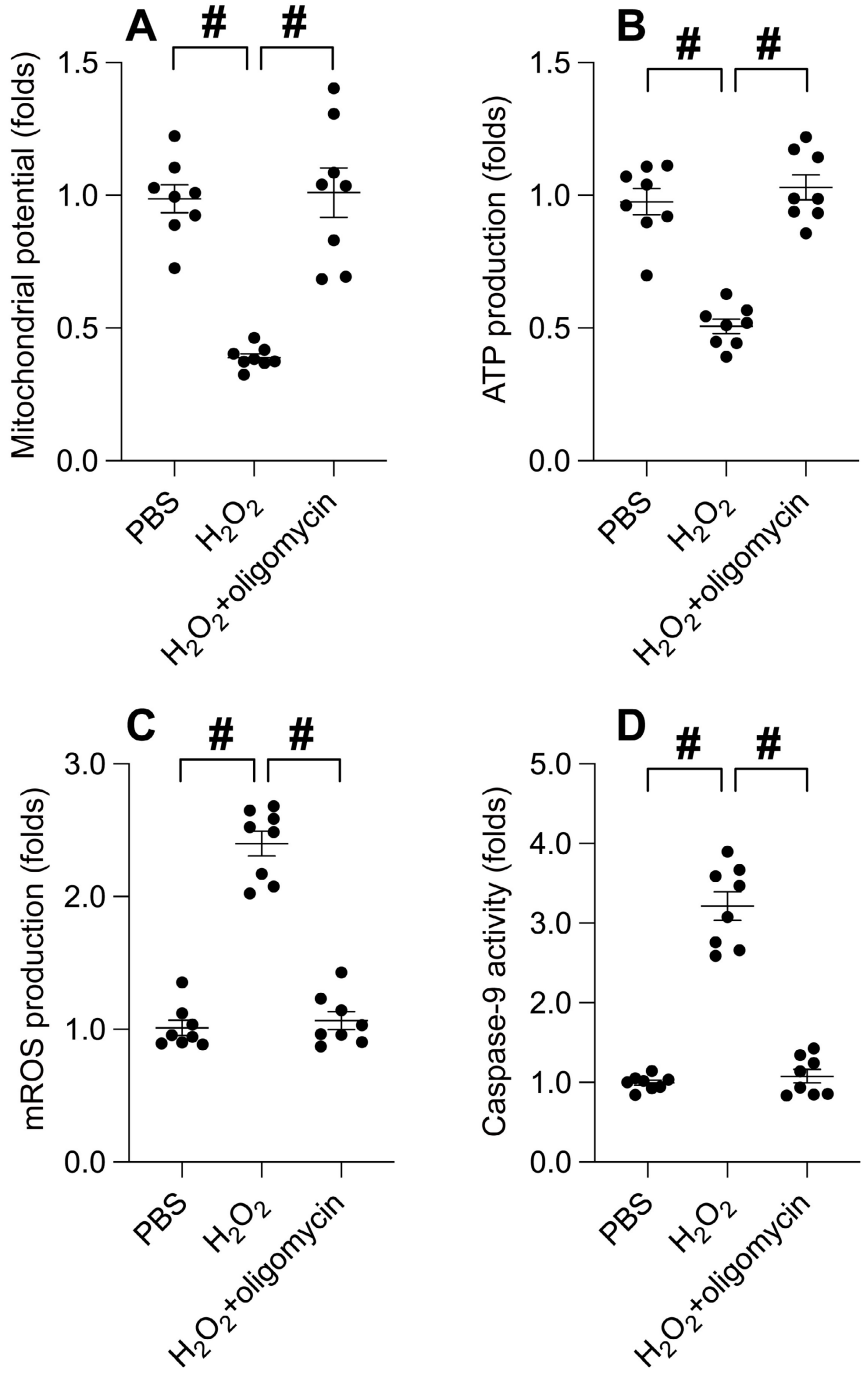
** UPRmt Activation Shields Mitochondria from Oxidative Stress-Induced Damage. A.** SUNE-1 cells were treated with hydrogen peroxide (0.3mM) to induce oxidative stress. Then, mitochondrial potential was determined by immunofluorescence. **B.** ATP production was measured through ELISA. **C.** Mitochondrial ROS were measured by immunofluorescence. **D.** ELISA kit was used to determine the activity of caspase-9. #p<0.05.

Subsequently, we delved into the potential correlation between UPRmt activation and the preservation of mitochondrial integrity under oxidative stress. Immunofluorescence analysis disclosed a disruption of mitochondrial membrane potential induced by hydrogen peroxide. Intriguingly, oligomycin treatment sustained the mitochondrial potential in the face of hydrogen peroxide exposure (Figure 4A). Moreover, ATP production, impeded by hydrogen peroxide, was notably conserved under oligomycin administration (Figure 4B). The generation of mitochondrial reactive oxygen species (ROS), a by-product of ATP synthesis, experienced a precipitous increase upon exposure to hydrogen peroxide (Figure 4C), which was attenuated to near-normal levels with oligomycin presence. Lastly, hydrogen peroxide incited an upsurge in caspase-9 activity (Figure 4D); however, this effect was counteracted by oligomycin. In aggregate, our findings suggest that UPRmt activation serves as a protective barrier for mitochondria against damage ensuing from oxidative stress.

### UPRmt activation mitigates cisplatin-induced cancer apoptosis

**Figure 5:**
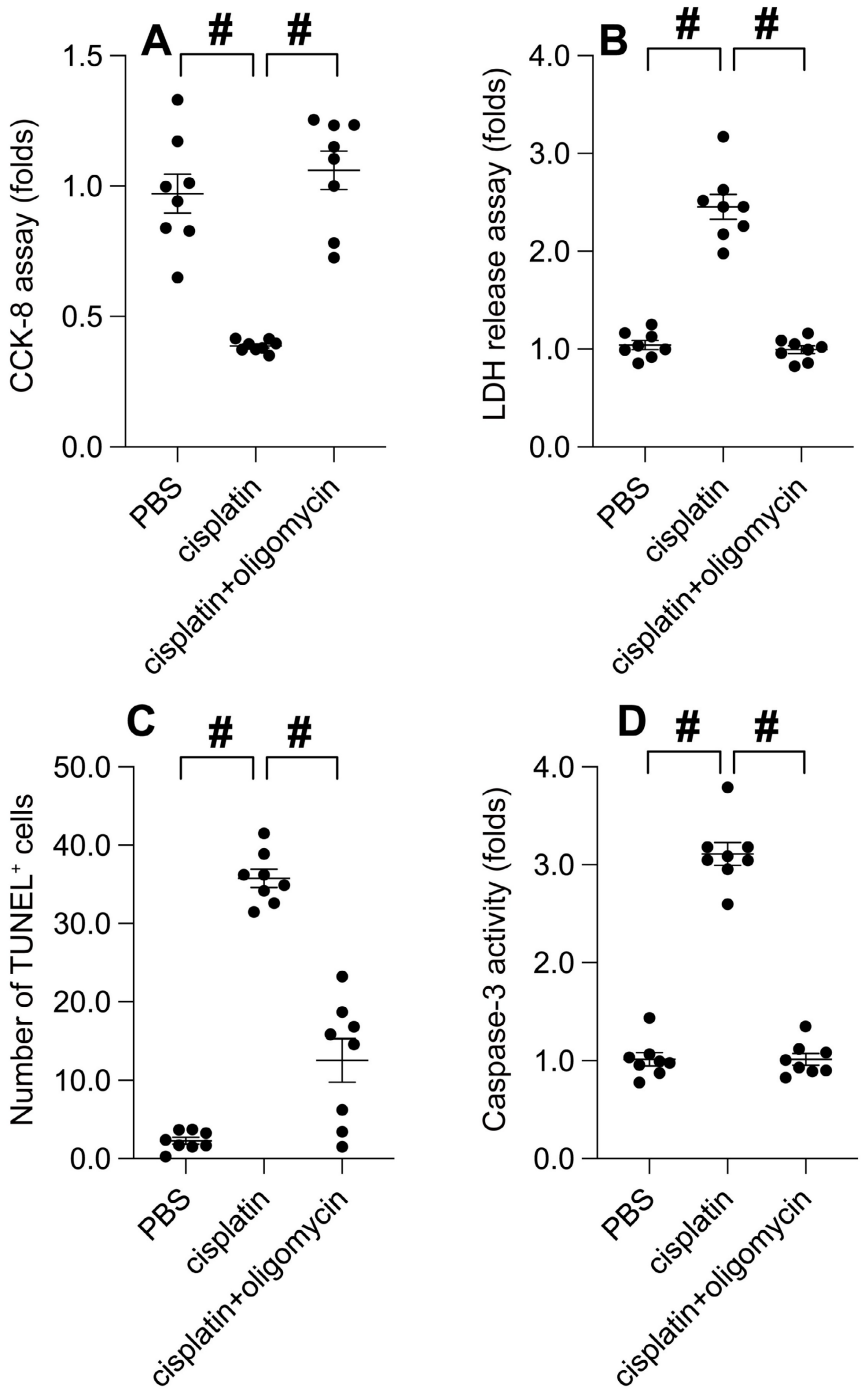
** UPRmt Activation Mitigates Cisplatin-Induced Cancer Apoptosis. A.** SUNE-1 cells were treated with cisplatin (5mM) to induce oxidative stress. Then, cell viability was determined by CCK-8 assay. **B.** LDH release assay was measured via an ELISA kit. **C.** The number of TUNEL positive cells was evaluated by immunofluorescence. **D.** ELISA kit was used to determine the activity of caspase-3. #p<0.05.

Chemotherapeutic agents, such as cisplatin, are renowned for their efficacy in curbing tumor progression by impacting mitochondrial function. Hence, we explored the potential role of UPRmt in cisplatin-mediated tumor suppression. Cisplatin treatment instigated a significant decrease in cell viability, as determined by the CCK-8 assay (Figure 5A). Concurrently, cisplatin incited the release of LDH (Figure 5B). Intriguingly, the administration of oligomycin notably preserved cell viability and curtailed LDH release in cisplatin-exposed SUNE-1 cells. TUNEL staining unveiled an increase in the proportion of TUNEL-positive cells post-cisplatin treatment (Figure 5C), whereas oligomycin treatment attenuated cell apoptosis. Analysis using an ELISA kit demonstrated a surge in caspase-3 activity in response to cisplatin treatment (Figure 5D). Notably, in SUNE-1 cells treated with oligomycin, cisplatin failed to elicit caspase-3 activation (Figure 5D). In summary, our findings underscore that UPRmt activation can mitigate cisplatin-induced cancer cell apoptosis.

### UPRmt activation mitigates cisplatin-induced mitochondrial dysfunction

**Figure 6:**
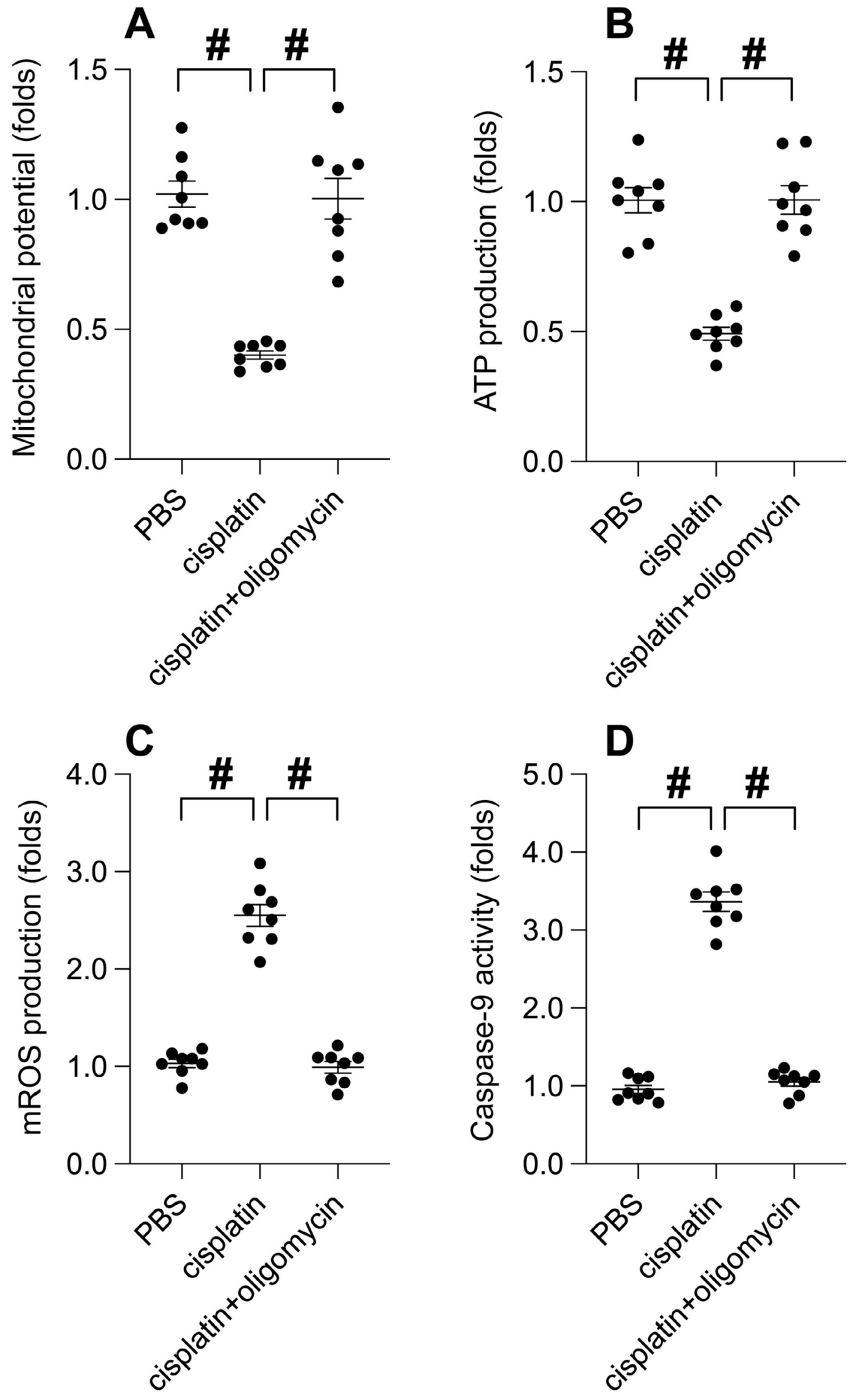
** UPRmt Activation Mitigates Cisplatin-Induced Mitochondrial Dysfunction. A.** SUNE-1 cells were treated with cisplatin (5mM) to induce oxidative stress. Then, mitochondrial potential was determined by immunofluorescence. **B.** ATP production was measured through ELISA. **C.** Mitochondrial ROS were measured by immunofluorescence. **D.** ELISA kit was used to determine the activity of caspase-9. #p<0.05.

At the molecular level, cisplatin treatment significantly precipitated mitochondrial dysfunction, as evidenced by a decrease in mitochondrial membrane potential. Intriguingly, UPRmt activation effectively conserved this potential (Figure 6A). Consequently, with the mitochondrial potential normalized, oligomycin treatment maintained mitochondrial ATP production despite cisplatin presence (Figure 6B). Furthermore, cisplatin incited the production of reactive oxygen species (ROS) in SUNE-1 cells, a phenomenon notably absent in cells treated with oligomycin (Figure 6C). Caspase-9, a key indicator of mitochondrial apoptosis, was activated by cisplatin. Remarkably, oligomycin treatment successfully thwarted the activation of caspase-9 (Figure 6D). In conclusion, our data emphasizes that UPRmt activation serves as a beneficial signal for preserving mitochondrial health in the face of cisplatin.

## Discussion

The activation of the mitochondrial unfolded protein response (UPRmt) has emerged as a crucial cellular defense mechanism against various forms of stress, particularly those that threaten mitochondrial integrity and function. Our study presents compelling evidence that UPRmt activation plays a significant role in protecting nasopharyngeal carcinoma cells from the deleterious effects of cisplatin, a widely used chemotherapeutic agent known for its cytotoxicity and induction of oxidative stress.

Cisplatin is renowned for its efficacy in cancer treatment but also notorious for its side effects, including nephrotoxicity and ototoxicity, largely due to its propensity to induce reactive oxygen species (ROS) and mitochondrial dysfunction 31, 32. In this context, our findings reveal that UPRmt serves as a cytoprotective pathway, mitigating cisplatin-induced mitochondrial damage and preserving cell survival. The study demonstrates that the inhibition of UPRmt, using an inhibitor such as ABESF, exacerbates cisplatin's cytotoxic effects, leading to increased LDH release, a surrogate marker of cell membrane integrity, and an elevation in TUNEL-positive cells, indicative of apoptosis. These effects are in stark contrast to those observed when UPRmt is activated by oligomycin treatment, which not only reduces LDH leakage but also diminishes the number of TUNEL-positive cells.

At the molecular level, our investigation into the impact of UPRmt on mitochondrial function has revealed that its activation preserves mitochondrial membrane potential and ATP production, even in the presence of cisplatin. This is particularly noteworthy given that ATP is essential for various cellular functions, and its depletion is a hallmark of cell death 33. The protective effects of UPRmt activation extend to attenuating the increase in ROS and inhibiting the activation of caspase-9, a key player in the apoptotic pathway.

The interplay between chemotherapeutic agents and cellular stress responses has been the subject of extensive research. While previous studies have elucidated various adaptive mechanisms that cells employ to counteract the cytotoxic effects of drugs like cisplatin, the role of the mitochondrial unfolded protein response (UPRmt) in cancer therapy has only recently begun to receive attention 34, 35. Our study contributes to this burgeoning field by providing novel insights into how UPRmt activation can shield cancer cells from cisplatin-induced mitochondrial dysfunction and apoptosis. Historically, the research on UPRmt has largely focused on its role in age-related diseases 36, 37 and metabolic disorders 38-40, where mitochondrial quality control is paramount. For instance, the activation of UPRmt has been shown to promote longevity in model organisms by ensuring mitochondrial proteostasis and function. However, the application of such findings to cancer biology has been limited.

In contrast to prior research, our study shifts the focus towards the protective role of UPRmt in the context of chemotherapeutic stress in cancer cells. This is a significant departure from the traditional view of UPRmt as merely a cellular defense mechanism against aging and disease. By showing that UPRmt can be co-opted by cancer cells to survive chemotherapy, our research adds a new dimension to the understanding of UPRmt, suggesting that it may also play a role in cancer cell resilience. Comparatively, earlier studies have primarily investigated the immediate cellular responses to chemotherapeutic agents, such as DNA damage response and apoptosis 41, 42. While these studies have been invaluable in understanding the primary mechanisms of drug action and resistance, they have not fully accounted for the role of the mitochondria-specific stress response, which is crucial given the mitochondria's role as the cellular powerhouse and a nexus for apoptotic signaling. Our findings also diverge from some previous reports in the literature that have hinted at a rather simplistic relationship between mitochondrial stress and cell death. We demonstrate a more nuanced scenario where UPRmt activation does not necessarily lead to cell demise but can, under certain conditions, promote survival. This is exemplified by our observations that oligomycin-induced UPRmt activation preserved mitochondrial membrane potential and ATP production, thereby conferring resistance to cisplatin's cytotoxicity. Moreover, our study sheds light on the potential therapeutic implications of modulating UPRmt. While the idea of targeting stress responses to enhance cancer therapy is not new, the specific targeting of UPRmt has not been extensively explored. Our research suggests that pharmacological modulation of UPRmt could be a viable strategy to augment the efficacy of cisplatin, adding to the growing list of novel therapeutic targets in oncology. In summary, our study stands out in the field by bridging the gap between mitochondrial biology and cancer therapy. It expands the current understanding of how cancer cells can harness cellular stress responses for survival, thereby providing a fresh perspective on the complex relationship between chemotherapy and cellular adaptive mechanisms. Further exploration of UPRmt in cancer could pave the way for the development of innovative strategies to enhance the therapeutic index of existing chemotherapeutic agents, ultimately leading to improved patient outcomes.

The implications of these findings are twofold. Firstly, they underscore the importance of UPRmt in maintaining mitochondrial homeostasis under stress conditions. This is consistent with the role of UPRmt in other pathophysiological contexts, such as neurodegenerative diseases and metabolic disorders, where mitochondrial function is compromised. Secondly, our data suggest that modulation of UPRmt could be a potential therapeutic strategy to enhance the efficacy of cisplatin by reducing its cytotoxicity to non-cancerous cells, thereby mitigating side effects. However, while our results are promising, they also raise several questions. For instance, the exact molecular pathways through which UPRmt exerts its protective effects remain to be fully elucidated. Furthermore, the long-term implications of UPRmt activation in cancer cells, particularly in terms of cancer progression and resistance to therapy, warrant further investigation.

In conclusion, the current study significantly advances our understanding of the role of UPRmt in cancer cell survival under chemotherapeutic stress. It highlights UPRmt as a double-edged sword that can both protect against cisplatin-induced cytotoxicity and potentially contribute to chemoresistance. Targeting UPRmt may therefore offer a novel approach to fine-tuning the therapeutic window of cisplatin, improving its efficacy while minimizing its collateral damage to healthy cells. Future studies delineating the precise mechanisms of UPRmt and its interaction with other cellular stress responses will be crucial for developing targeted therapies that can exploit this pathway for the benefit of cancer patients.

## Funding

This research is supported by the National Natural Science Foundation of China (Grant No. 82101227) and the Science and Technology Foundation of Guangzhou (Grant No. 2023A04J2091).
